# Building dignity at the bedside: A reflective clinical model for clinical encounters

**DOI:** 10.1017/S1478951525000343

**Published:** 2025-04-22

**Authors:** Miguel Julião, Loredana Buonaccorso

**Affiliations:** 1 Equipa Comunitária de Suporte em Cuidados Paliativos PalCo, ULS Amadora/Sintra, Amadora, Portugal; 2 Psycho-Oncology Unit, Azienda USL-IRCSS di Reggio Emilia, Reggio Emilia, Italy

**Keywords:** Dignity, personhood, clinical encounters, patient-centered care, reflective model, presence

Sometimes, simple ideas are born from simple moments.

During an observership, a resident approached the first author and said:

*“The concept of dignity is so complex that I don’t know how to apply it.*

*If I had an easier, more practical way, like a ‘sheet’ always in the pocket of my lab coat...”*

This sentence inspired the authors to write this personal reflection. It was that simple.

## Introduction

Clinical encounters are complex interactions that require more than just medical expertise; they demand a deep understanding of the patient as a person. Understanding the essential characteristics of a patient’s personhood enables healthcare providers to communicate and connect in a manner that is more aligned with the patient’s care goals.

**Figure 1.**
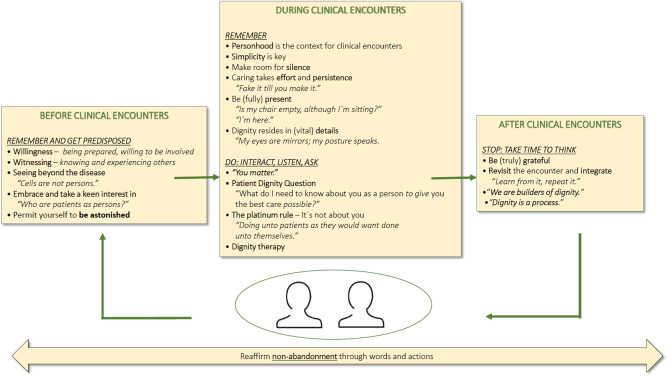
Dignity at the bedside: a reflective clinical model.

The model presented in this paper is divided into three interconnected phases: before, during, and after clinical encounters (Figure 1). Each phase is essential for building and maintaining dignity at the bedside. By preparing with curiosity and openness, engaging with active listening and empathy, and reflecting on the encounter afterward, healthcare providers can create a more meaningful and therapeutic relationship with the persons they care for.

We explore each phase in detail, providing practical guidance for healthcare providers who wish to integrate these principles into their practice. The goal is to propose a model of care that not only treats the person with the disease but affirms their inherent worth and dignity.

## Before clinical encounters: Preparing with curiosity and openness

The first phase of the model involves preparing for the clinical encounter with a sense of curiosity and openness. This preparation is essential for creating a therapeutic environment that prioritizes the patient’s personhood and dignity. In some settings, particularly in hospitals, there is often little time between visits. Approaching each interaction with curiosity helps healthcare providers stay focused, even in a few minutes, on understanding a patient’s unique health story and life. Curiosity is the first step in preparing for personalized care, reminding us that we may hear information we haven’t encountered before. Moreover, curiosity and openness reduce the risk of relying on automatic, standardized communication, fostering a foundation for dignity in care.

### Willingness and witnessing

Before meeting with a patient, healthcare providers should cultivate a sense of willingness – a readiness to be fully present and engaged in the encounter. This involves being prepared to witness the patient’s experiences, both medical and personal. As the model states, *“Witnessing – knowing and experiencing others”* is a key aspect of this preparation. By approaching the encounter with a sense of curiosity and openness, providers can create a space where patients feel seen and heard. For example, simple mindfulness practices, such as focusing on respiration, body, or one’s emotions, can help healthcare providers create relational space for the person (Buonaccorso et al. 2022).

### Seeing beyond the disease

One of the most important aspects of preparing for a clinical encounter is the ability to see beyond the disease. The model reminds us that *“Cells are not persons,”* emphasizing the need to view the patient as a whole person (Chochinov 2023), not just a collection of symptoms or a medical condition. This requires providers to embrace and take a keen interest in the question, *“Who are patients as persons?.”* By understanding the patient’s unique experiences, values, and needs, providers can tailor their care to address the person behind the illness. Additionally, being open to understanding the person living with the disease helps healthcare providers recognize the patient’s coping strategies and creates space for discussions beyond the illness, such as family, friends, work, and hobbies.

### Permitting yourself to be astonished

Finally, preparation involves allowing yourself to be astonished by the patient’s story and various aspects of their life (Julião et al. 2021a). The model encourages providers to *“permit yourself to be astonished,”* recognizing that each patient brings a unique and valuable perspective to the encounter. This sense of wonder and deep interest in the person before us can help providers approach the encounter with a sense of curiosity and openness, fostering a deeper connection with the patient. Preparing to listen to a life story, rather than just focusing on a disease, enables healthcare professionals to discover new, patient-specific ways of providing care. The biomedical paradigm emphasizes diagnosing and identifying interventions to “fix” the disease. However, “permitting yourself to be astonished” encourages awareness that we cannot predict the outcome of our interactions with a patient, helping us collaboratively build the clinical path together. This approach is rooted in “therapeutic humility” (Chochinov 2023), which involves letting go of the need to fix and embracing clinical ambiguity while trusting the relational process.

## During clinical encounters: Engaging with presence and dignity

The second phase of the model focuses on the clinical encounter itself, emphasizing the importance of presence, simplicity, and dignity in interactions with patients.

### Personhood as the context

The model reminds us that *“Personhood is the context for clinical encounters,”* emphasizing the need to view the patient as a whole person, not just a medical case. This requires providers to approach the encounter with a sense of full presence, ensuring that they are fully engaged with the person with the disease. The metaphor of the empty chair – *“Is my chair empty, although I’m sitting?”* – serves as a powerful reminder of the importance of true presence during clinical interactions.

We can be physically present with a patient but still be emotionally or mentally absent in the relationship. Recognizing when this happens is crucial to reclaiming our presence in the moment. This is why the model highlights the importance of taking a brief pause, even if only for a few moments, before engaging with the patient, to cultivate this awareness.

### Simplicity and silence

Simplicity is key during clinical encounters. Providers should strive to communicate clearly and concisely, avoiding unnecessary complexity or medical jargon. Often, medical language serves as a defense mechanism against the emotional climate that can arise when delivering bad news. Healthcare providers should be able to recognize this pattern and simplify communication to retrieve the necessary information. The model also emphasizes the importance of making room for silence, allowing patients the space to express themselves fully. Silence is more than the absence of words. Compassionate silence is a kind of communication between clinician and patient that fosters healing; and can be a powerful tool for fostering reflection and connection, helping providers gain a deeper understanding of the patient’s needs and concerns (Back et al. 2009).

### Caring takes effort and persistence

Caring for patients requires effort and persistence. The model encourages providers to *“fake it till you make it,”* recognizing that maintaining a sense of compassion and empathy can be challenging, especially in high-stress environments. However, by consistently practicing these behaviors, providers can create a more supportive and therapeutic care environment.

Caring takes effort and persistence, particularly in healthcare, where clinical encounters based on personhood demand as much dedication as being an “athlete.” Healthcare professionals must engage deeply with each patient, recognizing their unique needs, preferences, and emotional states, and tailor their approach accordingly. This level of personalized care requires not just clinical expertise, but a relentless commitment to understanding and supporting the whole person – body, mind, and spirit. Much like an athlete training for peak performance, healthcare providers must maintain their energy, focus, and resilience. The effort to continuously improve, adapt, and provide compassionate care in every encounter can be exhausting, but it is this persistence that allows healthcare professionals to create meaningful connections and significantly impact the lives of those they serve. Similar to athletes, healthcare providers should train themselves to emotionally navigate conversations with patients. Additionally, the care team and colleagues can provide valuable support in fostering a metacognitive approach (Buonaccorso et al. 2022).

### Dignity resides in the details

Dignity resides in the details of clinical encounters. The model reminds us that *“My eyes are mirrors; my posture speaks,”* emphasizing the importance of nonverbal communication in conveying respect and care. Providers should be mindful of their body language, eye contact, and tone of voice, ensuring that they are communicating dignity and respect in every interaction – the tone of care (Chochinov 2023). This is particularly important in situations of intimate dependency where patients may feel vulnerable or exposed.

### “You matter”

Dame Cicely Saunders was a pioneering figure in the field of hospice and palliative care, whose work transformed the way society approaches end-of-life. Her holistic approach emphasized not just the physical needs of terminally ill patients but also their emotional, social, and spiritual well-being. One of Saunders’ most famous quotes, *“You matter because you are you, and you matter to the last moment of your life” (Saunders C)*, encapsulates her philosophy of care. This phrase underscores the inherent dignity of every individual, affirming their worth regardless of their condition or prognosis. It challenges the notion that terminal illness diminishes a person’s value and conducts to being a burden, instead advocating for continued love, respect, and comfort throughout their final days. Through her work and words, Dame Saunders reshaped the global approach to dying, ensuring that patients are treated with kindness and empathy until the very end.

### The patient dignity question

The dignity question is a key concept in palliative and end-of-life care, stemming from research on dignity-conserving care. It asks, “What do I need to know about you as a person to give you the best care possible?” (Chochinov et al. 2015). This shifts focus from the disease to the individual, emphasizing their unique identity, values, and life story. It helps healthcare providers move beyond clinical symptoms to engage with the person, ensuring care aligns with what matters most to the individual. The dignity question challenges the depersonalized approach in medicine, recognizing patients’ intrinsic worth and guiding compassionate, individualized care. It encourages seeing patients as whole persons, deserving care that honors their humanity.

### The Platinum Rule

The Platinum Rule, introduced by Harvey Chochinov in 2022, guides clinical interactions with the principle: “Doing unto patients as they would want done unto themselves.” This rule emphasizes understanding and respecting each patient’s unique preferences and needs, tailoring care to their circumstances. Unlike the Golden Rule, which promotes treating others as you want to be treated, the Platinum Rule encourages treating others as they want to be treated. It highlights the importance of respecting each individual’s preferences, values, and experiences, especially in end-of-life care. Applying this rule fosters trust, ensures patients feel heard and respected, and improves care by addressing emotional, psychological, and spiritual needs in line with their beliefs.

### Dignity therapy

Dignity therapy is an innovative and compassionate approach to end-of-life care that focuses on preserving and enhancing the dignity of patients with terminal illness. It has been shown to reduce psychological distress, improve quality of life, and help patients approach death with greater peace and empowerment, allowing them to confront their mortality with a renewed sense of self and comfort. (Chochinov 2012; Julião et al. 2013). Dignity therapy focuses on helping individuals reflect on their life experiences, values, and the legacy they wish to leave behind, providing them with a sense of meaning and purpose in their final days.

## After clinical encounters: Reflecting and integrating

The final phase of the model involves reflecting on the clinical encounters and integrating the lessons learned into future practice.

### Taking time to think

After the encounter, providers should take time to reflect on the interaction, considering what went well and what could be improved. The model encourages providers to *“be truly grateful”* for the opportunity to care for the person, recognizing the privilege and responsibility of their role. This reflection can help providers gain a deeper understanding of the person’s needs and experiences, fostering continuous growth and improvement.

Building strong relationships between healthcare professionals and patients is crucial for effective care and requires time and persistence. Patience is important when overcoming barriers like emotional, cultural, or psychological hesitancy. Healthcare professionals also need to acknowledge their own emotions and practice self-awareness to avoid distractions and listen attentively to patients, fostering deeper care (Sansó et al. 2015).

### Revisiting and integrating the encounter

Reflection also involves revisiting the encounter and integrating the lessons learned into future practice. The model encourages providers to *“learn from it, repeat it,”* recognizing that each encounter offers valuable insights that can inform future interactions. Like the athlete who revises each of his tournaments and trainings, providers are called to integrate these lessons into their practice so they can continuously improve their ability to provide patient-centered care.

### Building dignity is a process

Finally, reflection involves recognizing that *“dignity is a process”* and that providers are *“builders of dignity.”* This process requires ongoing effort and commitment, as providers work to create a care environment that affirms the inherent worth and dignity of every patient. By embracing this process, providers can create a more compassionate and effective care environment, fostering *healing* and connection with their patients.

### Non-abandonment

Non-abandonment is a foundational ethical principle in healthcare, reflecting the commitment of healthcare professionals to stand by their patients through every stage of their illness, particularly during moments of vulnerability, uncertainty, or suffering. This principle is especially vital in fields such as palliative care, chronic disease management, and end-of-life care, where patients often face not only physical pain but also emotional and existential distress. Non-abandonment ensures that patients feel supported and valued, even when curative treatments are no longer viable. By remaining present and engaged, healthcare professionals provide a sense of stability and trust, which can significantly alleviate feelings of fear, or isolation leading to the desire for death (Chochinov et al. 1995; Julião et al. 2013, 2021b). Non-abandonment requires healthcare professionals to go beyond clinical tasks and to actively listen, empathize, and respond to the emotional, social, and spiritual needs of their patients, through words and actions. This approach is particularly meaningful in situations where the focus shifts from curing to caring, such as in terminal illness, advanced chronic conditions or acute and intensive care. “Being present with self-awareness” means that in every moment of care assistance, it is possible to support an image of the patient among healthcare providers that restores dignity and gives meaning to the care relationship, even when this image is not consciously recognized by the patient (Buonaccorso L, De Panfilis L, Chochinov 2023).

## Conclusion

The model presented emphasizes the importance of personhood, dignity, and presence in clinical encounters. By preparing with curiosity and openness, engaging with presence and dignity, and reflecting on the encounter afterward, healthcare providers can create a more compassionate and effective care environment. This approach might not only improve patients’ outcomes but also enriches the practice of healthcare, making it a more meaningful and fulfilling profession. Moreover, healthcare professionals must take care of their health and well-being to support their competence in caring for patients.

In conclusion, like with the persistent “athlete,” building dignity at the bedside requires a commitment to continuous learning and growth. By integrating the principles outlined in this model into their practice, we hope healthcare providers can create a more patient-centered approach to care that honors the individuality and dignity of every patient.
